# Local radiotherapy in extensive-stage small-cell lung cancer sustainably boosts the clinical benefit of first-line immunotherapy: a case report

**DOI:** 10.3389/fimmu.2024.1493740

**Published:** 2024-11-01

**Authors:** Hongming Wang, Nuoni Wang, Shiyan Li, Yangfeng Du, Tao Wu, Wei Tian, Wen Dong, Xiaoyang Liu, Yan Zhang, Jiang Zheng, Zemin Xiao, Zhijun Wu

**Affiliations:** ^1^ Department of Oncology, Changde Hospital, Xiangya School of Medicine, Central South University (The First People’s Hospital of Changde City), Changde, China; ^2^ Department of Electrocardiogram and Physiology, Changde Hospital, Xiangya School of Medicine, Central South University (The First People’s Hospital of Changde City), Changde, China

**Keywords:** local radiotherapy, extensive-stage small-cell lung cancer, clinical benefit, immunotherapy, case report

## Abstract

**Background:**

Extensive-stage small-cell lung cancer (ES-SCLC) has a dismal prognosis owing to its high aggressiveness, rapid drug resistance, and early metastasis. ES-SCLC responds well to first-line chemotherapy, and chemotherapy coupled with immunotherapy can further improve overall survival. However, the long-term survival of patients remains unsatisfactory because of its high recurrence rate and the poor efficacy of second-line treatment. Although local radiotherapy is an important component of the overall treatment for ES-SCLC, its value in the age of immunotherapy remains controversial.

**Case description:**

A 54-year-old male with ES-SCLC achieved a complete response (CR), as determined using enhanced computed tomography (CT) after four cycles of immunochemotherapy (serplulimab, carboplatin, and etoposide). Whole-body positron emission tomography-CT was performed during maintenance treatment with serplulimab, which showed primary lung, liver, and bone metastatic lesions with CR. However, several mediastinal lymph nodes exhibited glucose metabolism uptake, and new lesions appeared on the head. The patient underwent palliative radiotherapy of the head and consolidative thoracic radiotherapy of the chest and continued maintenance treatment with serplulimab. Subsequent magnetic resonance imaging of the head suggested good control of metastatic lesions (CR). The patient received first-line immunotherapy for approximately 20 months.

**Conclusions:**

This report presents a patient with ES-SCLC who underwent local radiotherapy in addition to serplulimab as maintenance therapy. Although the programmed death-ligand 1 (PD-L1) expression level was negative and a PD-1 inhibitor instead of a PD-L1 inhibitor was used, the patient did not experience significant pneumonia during treatment, and the efficacy of the current treatment was evident. This treatment model warrants further clinical investigation.

## 1 Introduction

Small-cell lung cancer (SCLC) is the most aggressive subtype of lung cancer, accounting for approximately 15% of all cases. It is a highly malignant neuroendocrine tumor, and smoking has been identified as the biggest risk factor. Surgery, radiotherapy, and chemotherapy are the three main treatments for SCLC; however, extensive-stage SCLC (ES-SCLC) is not eligible for radical surgery and concurrent chemoradiotherapy because of late staging. The median overall survival (mOS) of patients with SCLC without active treatment is only 2–4 months (1, 2). Over the past few decades, platinum-based chemotherapy has become the preferred first-line therapy for ES-SCLC; however, the mOS remains only 9–10 months, and the 2-year OS rate is <5% (3).

With the development of immunotherapy, treatment outcomes for SCLC have improved. Immunochemotherapy has replaced simple chemotherapy as the new first-line treatment standard for ES-SCLC (4). Compared with non-SCLC, which responds relatively well to immunotherapy, the overall effectiveness of immune checkpoint inhibitors in ES-SCLC is still not optimistic, which may be related to the immunosuppressive features of SCLC, such as lower CD8+ T-cell infiltration, expression level of major histocompatibility complex class I molecules, and expression level of programmed death-ligand 1 (PD-L1) (5, 6). Although most patients with ES-SCLC respond well to initial first-line immunochemotherapy, they rapidly relapse and require further treatment; however, backline therapeutic choices are scarce and frequently less effective. Therefore, effective combination therapy options are urgently required to delay SCLC progression. Radiotherapy, in combination with immunotherapy, is a promising treatment modality. Studies have shown that radiotherapy not only directly attacks cancer cells but also induces their immunogenic death, remodels the tumor immune microenvironment by upregulating tumor antigens, promoting their release and presentation, and improves the ratio of tumor-infiltrating lymphocytes to immunosuppressive cells, thus synergistically combating tumors with immune checkpoint inhibitors (7, 8). Whether combined radiotherapy can reverse the immune desert state of SCLC and further enhance its curative effect has become a research hotspot.

Here, we report that adding local radiotherapy during the immunotherapy maintenance period in a patient with ES-SCLC effectively boosted the clinical benefit of first-line immunotherapy and is expected to substantially prolong the overall survival with a favorable safety profile.

## 2 Case presentation

This study involved a human participant and was approved by the Ethics Committee and the Institutional Review Board of Changde Hospital, Xiangya School of Medicine, Central South University (Approval no: 2024-244-01; date: [September 6, 2024]). The study was conducted in accordance with local legislation and institutional requirements. The patient provided written informed consent to participate in the study and for the publication of any potentially identifiable images or data included in this article. This study complies with the standard reporting guidelines (CARE).

**Figure 1:**
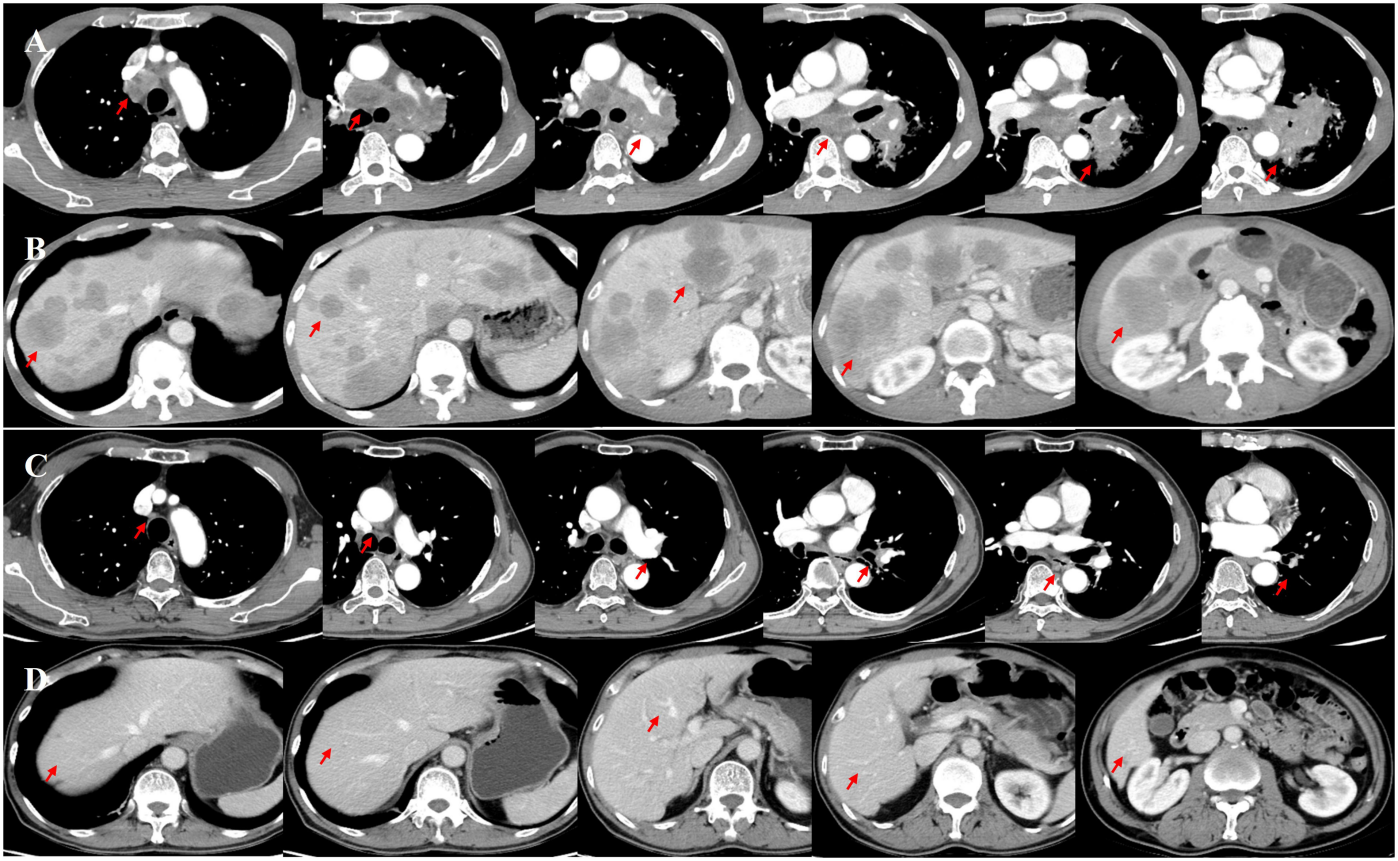
Enhanced computed tomography (CT) of the chest and abdomen was initially performed on December 27, 2022 **(A, B)**. After four cycles of immunochemotherapy, a follow-up enhanced CT was performed on April 22, 2023, showing the disappearance of the primary lesion in the left lung, notable shrinkage of the left lung hilar and mediastinal lymph nodes (short axis <10 mm), and absence of obvious metastatic lesions in the liver **(C, D)**.

On December 26, 2022, a 54-year-old Chinese male presented to our hospital with complaints of cough, blood-stained sputum, a hoarse voice, shortness of breath after activity, and pain in the posterior lumbar back and right epigastrium lasting >1 month. The patient’s general condition was poor; however, there were no symptoms of discomfort such as chills, fever, dizziness, headache, nausea, or vomiting. He was previously healthy but often stayed up late. The patient did not have any infectious or genetic diseases, nor did they have a family history of cancer. However, he did have a history of excessive drinking (>40 years) and smoking (>30 years). Liver palpation indicated that the lower edge of the tumescent liver was located two transverse fingers below the ribs, and no other evident physical findings were observed. Contrast-enhanced computed tomography (CT) of the chest and abdomen revealed a central-type tumor in the lower lobe of the left lung with obstructive pneumonia, invasion of the left pulmonary artery and vein, multiple mediastinal regional lymph nodes, and liver metastases (**Figures 1A, B**). A whole-body bone scan revealed numerous bone metastases throughout the body, including in the left scapula, sternum, left ribs, cervical vertebrae, thoracic vertebrae, lumbar vertebrae, and pelvis. No brain metastases were detected on magnetic resonance imaging (MRI). Bronchoscopic biopsy revealed SCLC with negative PD-L1 expression (**Supplementary Figure 1**). A diagnosis of ES-SCLC, cT4N3M1c2, stage IVB (International Association for the Study of Lung Cancer 9th Edition) was confirmed.

**Figure 2:**
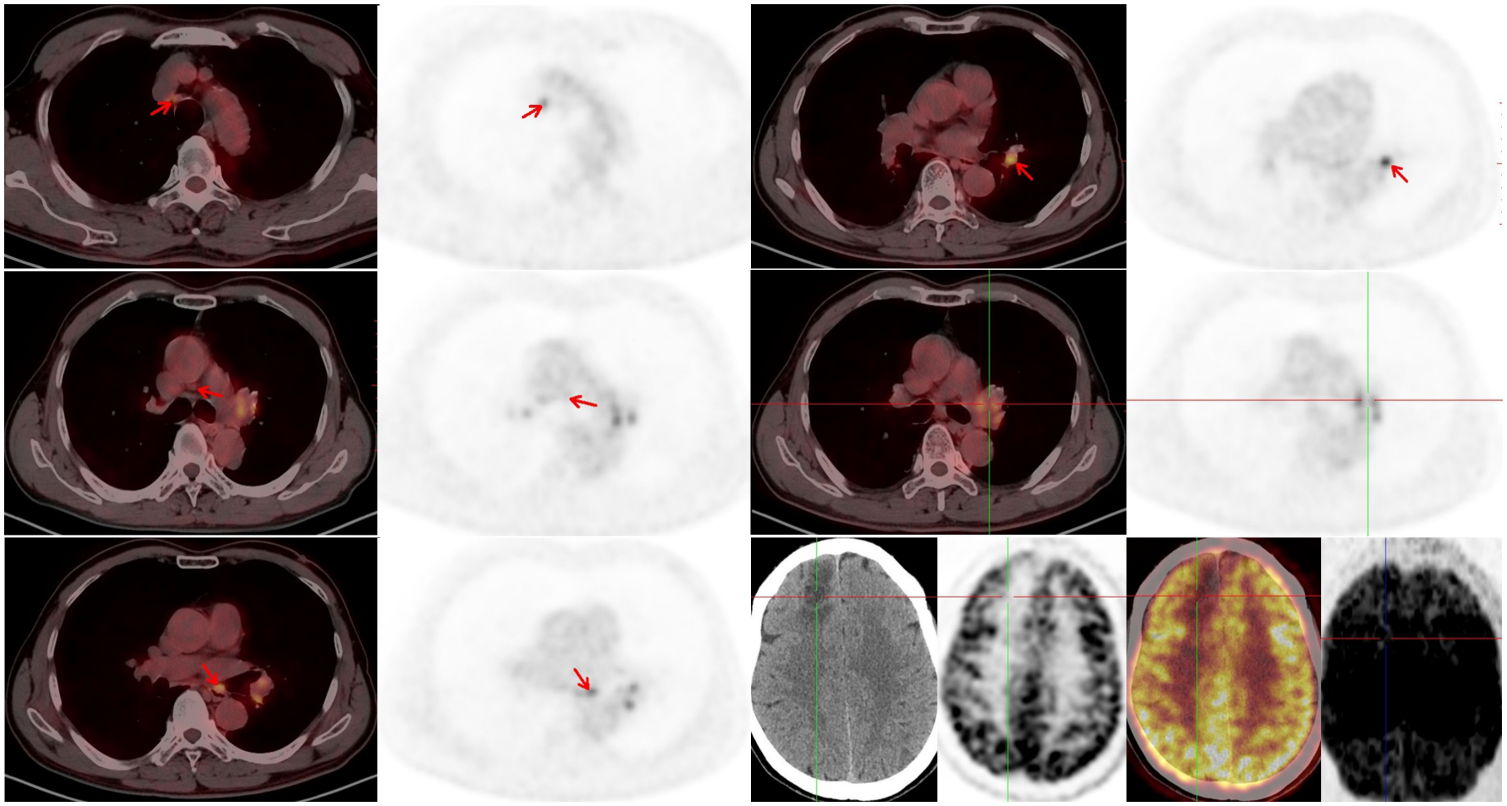
Positron emission tomography (PET)-computed tomography (CT) was performed on October 27, 2023. A residual tumor in the left lung hilar and mediastinal lymph nodes and new metastatic lesions in the brain can be observed.

**Figure 3:**
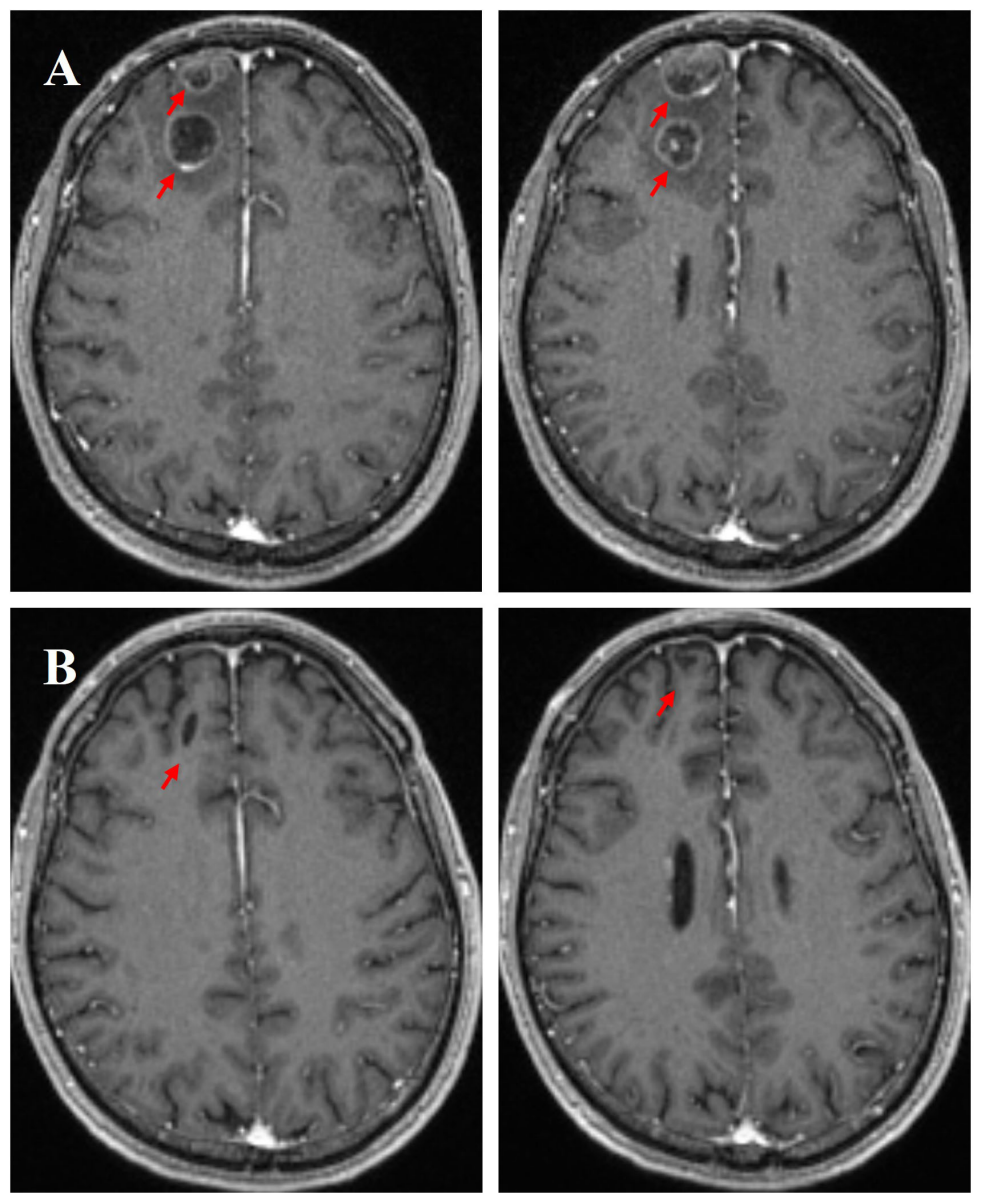
Enhanced magnetic resonance imaging (MRI) scans of the brain on October 28, 2023, further confirmed new brain metastases **(A)**. Following palliative radiotherapy, an MRI evaluation of the brain metastatic lesions was performed on February 23, 2024, showing a complete response (CR) **(B)**.

**Figure 4:**
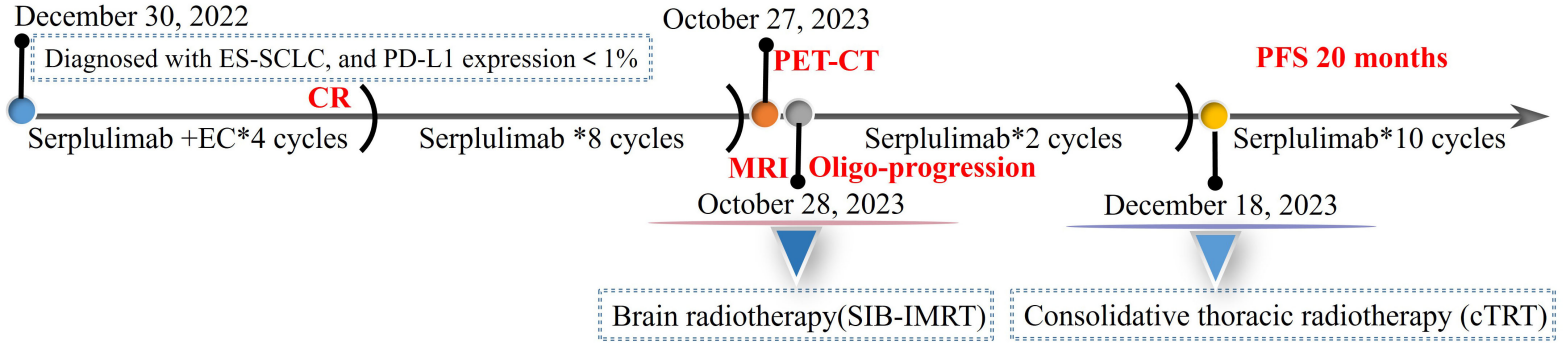
The course of disease and treatment. ES-SCLC, extensive-stage small-cell lung cancer; PD-L1, programmed death-ligand 1; EC, etoposide and carboplatin; CR, complete response; PET-CT, positron emission tomography-computed tomography; MRI, magnetic resonance imaging; SIB-IMRT, simultaneous integrated boost intensity-modulated radiation therapy; PFS, progression-free survival.

Immunochemotherapy is the preferred first-line treatment regimen. From January 05 to March 28, 2023, the patient was administered four cycles of serplulimab (4.5 mg/kg, day 1), carboplatin (AUC 5, day 1), and etoposide (100 mg/m^2^, day 1–3); he experienced moderate gastrointestinal reactions and no other adverse events. The therapeutic effect evaluated using CT was a complete response (CR) (**Figures 1C, D**). He was started on serplulimab 200 mg (4.5 mg/kg) administered every 21 days as a maintenance regimen on April 23, 2023. A whole-body positron emission tomography (PET)-CT review was performed on October 27, 2023 (**Figure 2**), which showed the following: increased glycometabolism in the left lung hilar and mediastinal (group 2R/4R/8) lymph nodes (maximum standardized uptake volume [SUVmax]: 6.9), suggesting that local tumor cells were still active (partial response, PR); no glucometabolic concentrations in the liver and whole-body bone, suggesting that local tumor cells were inactive (CR); and two new annular nodules in the right frontal lobe, not accompanied by increased glucose metabolism, which were considered as brain metastases. Further brain MRI confirmed the diagnosis of brain metastases (oligo-progression) (**Figure 3A**).The patient underwent palliative radiotherapy (simultaneous integrated boost intensity-modulated radiation therapy [SIB-IMRT]) for the head lesions on October 31, 2023, with a planning gross target volume dose of 45 Gy in 10 fractions (fx) and planning target volume dose of 30 Gy/10 fx (once a day). He also underwent consolidative thoracic radiotherapy (cTRT) on December 18, 2023, with a planning target volume dose of 39 Gy/26 fx (twice a day). No significant adverse effects were observed. On February 23, 2024, a follow-up MRI showed CR of the metastatic head lesions (**Figure 3B**). The patient underwent strontium (Sr-89) chloride radionuclide therapy on June 30, 2023, January 29, 2024, and August 20, 2024, respectively, and bisphosphonate therapy once a month to reduce the risk of pathological fracture. The patient continued to receive maintenance therapy with serplulimab, and first-line immunotherapy was administered for up to 20 months. Patient compliance and tolerability were good. At the most recent follow-up examination, the patient exhibited no signs of deterioration, no immunotherapy-related adverse effects, and a high quality of life. We plan to maintain immunotherapy until disease progression or the occurrence of intolerable toxicity. The patient’s treatment course is depicted in **Figure 4**.

## 3 Discussion

The era of immunotherapy for SCLC has dawned with the success of the IMPOWER133 and CASPIAN phase 3 clinical trials for ES-SCLC. However, in the immunotherapy groups in these two studies, the median progression-free survival (mPFS) was approximately 5 months, whereas the mOS was only 12–13 months (9, 10). The ASTRUM-005, EXTENTORCH, and RATIONALE-312 trials with PD-1 inhibitors followed suit with positive results, showing that the mOS significantly exceeded that with atezolizumab and durvalumab (11–13). However, the overall survival benefits for patients have reached a bottleneck. Thus, there is a need to screen potential beneficiaries of immunotherapy, seek new therapeutic modalities and tactics to further increase the efficacy of first-line immunotherapy, and ultimately enhance the long-term survival outcomes in patients with ES-SCLC.

Radiotherapy and immunotherapy are mechanistically well synergized; however, cTRT was not authorized in any of these studies, and the advantages and safety of combining immunotherapy with thoracic radiotherapy (TRT) remain controversial. In an era of chemotherapy, TRT coupled with prophylactic cranial irradiation has reduced the incidence of intrathoracic recurrence by 50% and increased the 2-year overall survival rate (13% vs. 3%, P = 0.004) in patients with ES-SCLC who achieved PR or CR following chemotherapy (14). An analysis of the progression patterns in the IMpower133 study revealed that the predominant progression in both arms occurred in existing lesions, especially in the lung and lymph nodes (15). This indicates that TRT after first-line immunotherapy may be beneficial and that consolidation radiotherapy for primary and metastatic lesions may further improve the first-line mPFS and overall prognosis of patients with ES-SCLC after immunochemotherapy. PD-1 inhibitors are associated with a greater risk of pneumonia during first-line ES-SCLC treatment than PD-L1 inhibitors (16). However, in the present case, the patient did not develop pneumonia of any grade after TRT and PD-1 immunomaintenance therapy. We did not specifically analyze the SCLC molecular subtype; we only confirmed that the PD-L1 expression level was negative. Although the patient’s condition was well-controlled using immunochemotherapy, brain radiotherapy (SIB-IMRT), and cTRT, we are still pondering whether the patient’s brain metastasis could have been avoided, thus further improving the patient’s prognosis by advancing the treatment time with cTRT and prophylactic cranial irradiation. In addition, we appreciate the importance of PET-CT examination before local radiotherapy, which can facilitate effective evaluation of the patient’s tumor control status and provide guidance for accurately delineating the subsequent radiotherapy target volume. Owing to the high cost, the patient did not undergo PET-CT for metabolic assessment of the mediastinal lymph nodes following TRT. Phase I/II prospective and retrospective studies have provided preliminary evidence for the safety and effectiveness of radiotherapy and immunotherapy in patients with ES-SCLC (17–24). However, further investigations of the optimal dose, segmentation pattern, and intervention time of TRT for ES-SCLC in the context of immunotherapy are required. Currently, phase III clinical studies on first-line immunotherapy combined with TRT for patients with ES-SCLC are underway (NCT04028050; NCT05223647; NCT04402788), and the results of this research are expected to be of great clinical significance.

Anti-angiogenic drugs have been used as the first-line treatment for various tumors; however, their application in the first-line therapy of ES-SCLC is still being explored. Anti-angiogenic drugs modulate the tumor immune microenvironment and synergize with immunotherapy. The ETER701 study showed that a four-drug combination regimen (benmelstobart, anlotinib, and chemotherapy) in ES-SCLC resulted in an mPFS of up to 6.9 months and the longest mOS (19.3 months) ever documented in the registry trials to date (25). At the 2024 American Society of Clinical Oncology annual meeting, a preliminary analysis of the BEAT-SC study showed that it reached its primary endpoint of investigator-assessed progression-free survival (PFS). Bevacizumab combined with atezolizumab + carboplatin or cisplatin + etoposide significantly prolonged the median investigator-assessed PFS when compared with the standard immunotherapy (5.7 months vs. 4.4 months, *P* = 0.0060); mOS data are currently unavailable (26). The phase Ib/II trial of surufatinib, toripalimab, and the etoposide and cisplatin (EP) combination for the first-line therapy of ES-SCLC demonstrated its therapeutic potential (overall response rate: 97.1%; disease control rate: 100%), and subsequent survival results are highly anticipated (27). Preliminary results from a phase Ib clinical trial of the novel PD-1/VEGF biospecific antibody AK112 in conjunction with the EP regimen for the first-line treatment option of ES-SCLC showed promising antitumor efficacy and survival benefits with a manageable safety profile (28). Immunochemotherapy coupled with anti-angiogenic drugs is another potential strategy for improving the first-line treatment efficacy in patients with ES-SCLC. However, we also need to further explore the beneficiaries and safety of anti-angiogenic drugs combined with immunochemotherapy, optimize the drug maintenance dose and duration, and make the treatment more precise and individualized. Follow-up studies are expected to provide more evidence to maximize the clinical benefits for patients with ES-SCLC.

Based on immunochemotherapy, novel first-line treatment modes for ES-SCLC are being actively explored. Clinical studies of immunochemotherapy combined with tarlatamab (AMG757), a DLL3-targeting half-life extended bispecific T-cell engager (HLE BiTE^®^), tifcemalimab (JS004), a humanized IgG4 monoclonal antibody against B and T lymphocyte attenuator, and autologous natural killer cells for the first-line treatment of ES-SCLC are currently ongoing (29–31). Clinical studies are also underway for the addition of the novel chemotherapy drug lurbinectedin, a poly-adenosine diphosphate ribose polymerase inhibitor, or bomedemstat, a lysine-specific demethylase 1 inhibitor (NCT05191797), to first-line immunomaintenance therapy in ES-SCLC (32–34). The results of these studies are promising and provide valuable medical evidence and treatment options in clinical settings.

In summary, our case revealed the notable efficacy and safety of a PD-1 inhibitor (serplulimab) combined with chemotherapy and local radiotherapy for ES-SCLC with negative PD-L1 expression. In the first-line treatment of ES-SCLC, combining radiotherapy with immunotherapy is another major issue that requires further investigation. These questions and debates will guide the way forward for clinical trials, which will ultimately lead to breakthroughs in the quality of life and survival benefits of patients with ES-SCLC.

## Data Availability

The original contributions presented in the study are included in the article/**Supplementary Material**. Further inquiries can be directed to the corresponding authors.
