# Supplementary material for: Local radiotherapy in extensive-stage small-cell lung cancer sustainably boosts the clinical benefit of first-line immunotherapy: a case report
Source: Front Immunol. 2024 Nov 1;15:1493740. doi: 10.3389/fimmu.2024.1493740 (PMC11563787; doi:10.3389/fimmu.2024.1493740)

## Slide 1
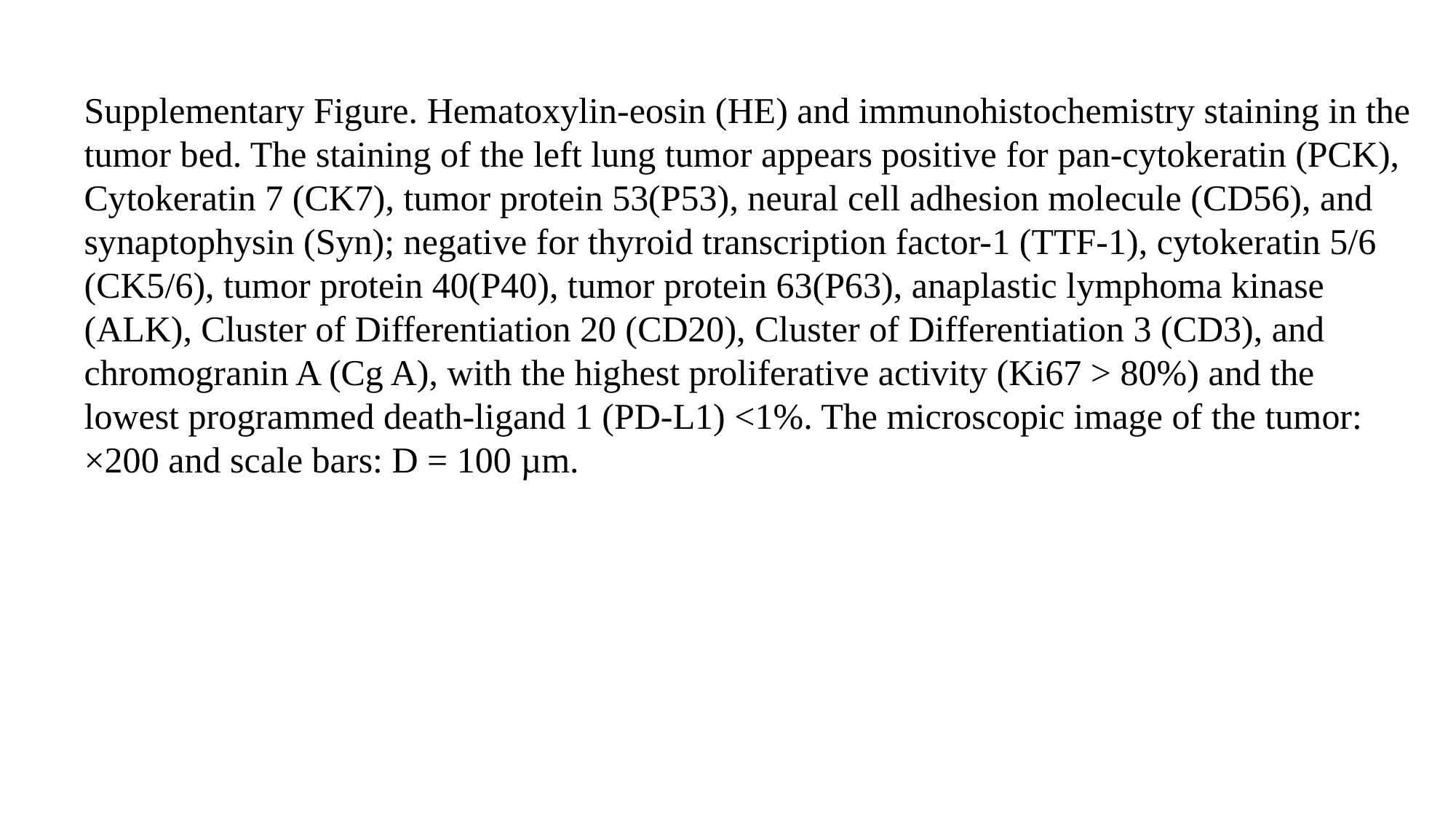

Supplementary Figure. Hematoxylin-eosin (HE) and immunohistochemistry staining in the tumor bed. The staining of the left lung tumor appears positive for pan-cytokeratin (PCK), Cytokeratin 7 (CK7), tumor protein 53(P53), neural cell adhesion molecule (CD56), and synaptophysin (Syn); negative for thyroid transcription factor-1 (TTF-1), cytokeratin 5/6 (CK5/6), tumor protein 40(P40), tumor protein 63(P63), anaplastic lymphoma kinase (ALK), Cluster of Differentiation 20 (CD20), Cluster of Differentiation 3 (CD3), and chromogranin A (Cg A), with the highest proliferative activity (Ki67 > 80%) and the lowest programmed death-ligand 1 (PD-L1) <1%. The microscopic image of the tumor: ×200 and scale bars: D = 100 µm.
A

## Slide 2
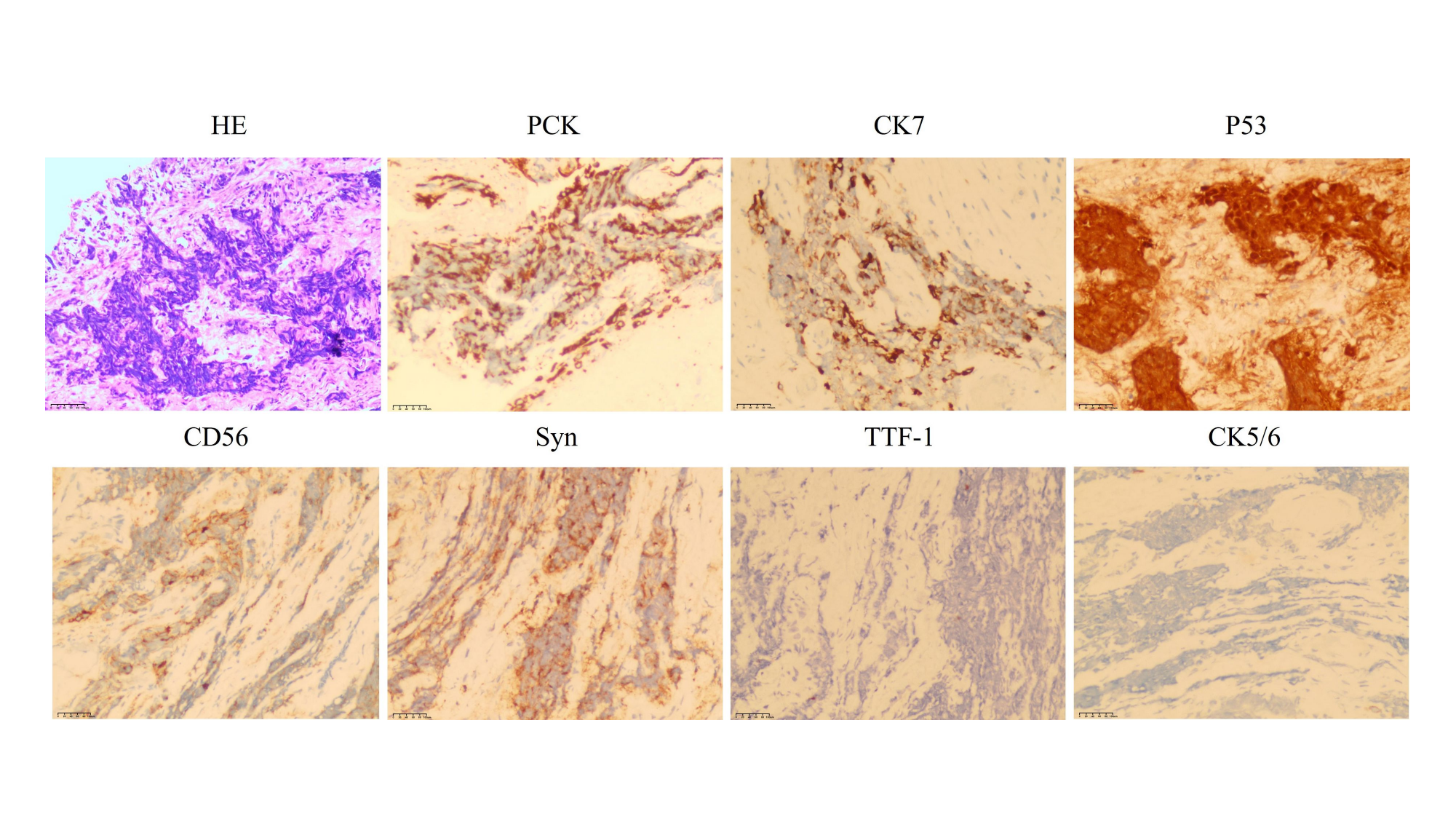

## Slide 3
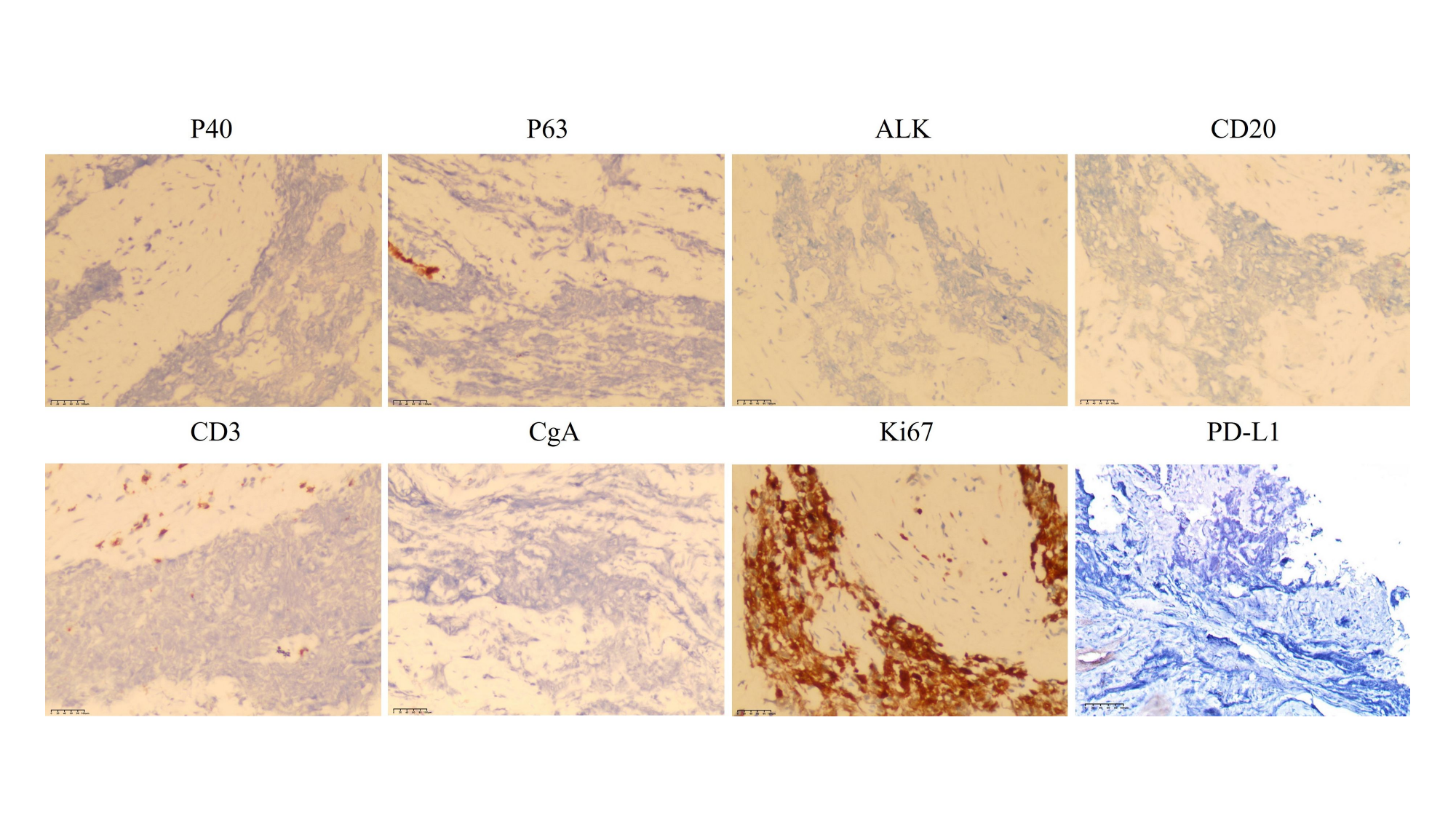

Supplement: Supplementary file 1 [file Presentation1.pptx]
